# Tyrosyl-DNA phosphodiesterases are involved in mutagenic events at a ribonucleotide embedded into DNA in human cells

**DOI:** 10.1371/journal.pone.0244790

**Published:** 2020-12-31

**Authors:** Ayuna Takeishi, Hiroyuki Kogashi, Mizuki Odagiri, Hiroyuki Sasanuma, Shunichi Takeda, Manabu Yasui, Masamitsu Honma, Tetsuya Suzuki, Hiroyuki Kamiya, Kaoru Sugasawa, Kiyoe Ura, Akira Sassa

**Affiliations:** 1 Department of Biology, Graduate School of Science, Chiba University, Chiba, Japan; 2 Department of Radiation Genetics, Graduate School of Medicine, Kyoto University, Yoshida Konoe, Sakyo-ku, Kyoto, Japan; 3 Division of Genetics and Mutagenesis, National Institute of Health Sciences, Setagaya-ku, Tokyo, Japan; 4 Graduate School of Biomedical and Health Sciences, Hiroshima University; Minami-ku, Hiroshima, Japan; 5 Biosignal Research Center, Kobe University, Kobe, Japan; University of South Alabama Mitchell Cancer Institute, UNITED STATES

## Abstract

Ribonucleoside triphosphates are often incorporated into genomic DNA during DNA replication. The accumulation of unrepaired ribonucleotides is associated with genomic instability, which is mediated by DNA topoisomerase 1 (Top1) processing of embedded ribonucleotides. The cleavage initiated by Top1 at the site of a ribonucleotide leads to the formation of a Top1-DNA cleavage complex (Top1cc), occasionally resulting in a DNA double-strand break (DSB). In humans, tyrosyl-DNA phosphodiesterases (TDPs) are essential repair enzymes that resolve the trapped Top1cc followed by downstream repair factors. However, there is limited cellular evidence of the involvement of TDPs in the processing of incorporated ribonucleotides in mammals. We assessed the role of TDPs in mutagenesis induced by a single ribonucleotide embedded into DNA. A *supF* shuttle vector site-specifically containing a single riboguanosine (rG) was introduced into the human lymphoblastoid TK6 cell line and its *TDP1*-, *TDP2*-, and *TDP1*/*TDP2*-deficient derivatives. *TDP1* and *TDP2* insufficiency remarkably decreased the mutant frequency caused by an embedded rG. The ratio of large deletion mutations induced by rG was also substantially lower in *TDP1*/*TDP2*-deficient cells than wild-type cells. Furthermore, the disruption of TDPs reduced the length of rG-mediated large deletion mutations. The recovery ratio of the propagated plasmid was also increased in *TDP1*/*TDP2*-deficient cells after the transfection of the shuttle vector containing rG. The results suggest that TDPs-mediated ribonucleotide processing cascade leads to unfavorable consequences, whereas in the absence of these repair factors, a more error-free processing pathway might function to suppress the ribonucleotide-induced mutagenesis. Furthermore, base substitution mutations at sites outside the position of rG were detected in the *supF* gene via a TDPs-independent mechanism. Overall, we provide new insights into the mechanism of mutagenesis induced by an embedded ribonucleotide in mammalian cells, which may lead to the fatal phenotype in the ribonucleotide excision repair deficiency.

## Introduction

In eukaryotic cells, ribonucleoside triphosphates are occasionally incorporated by DNA polymerases (Pols) during DNA replication [1]. It is estimated that more than 1,000,000 ribonucleotide molecules are embedded into genomic DNA during single-round replication in mammals [2]. The transient incorporation of ribonucleotides by Pols is beneficial for efficient DNA repair. For example, genomic ribonucleotides serve as a signal for strand discrimination during DNA mismatch repair [3,4]. Ribonucleotide incorporation during DNA double-strand break (DSB) repair also promotes efficient DNA ligation reactions [5]. However, dysfunction of the appropriate repair pathway for embedded ribonucleotides causes the accumulation of ribonucleotides, leading to genomic instability and resulting in tumorigenesis and disease [6–8]. Therefore, genomic ribonucleotide incorporation is a double-edged sword for genome integrity.

Embedded ribonucleotides are primarily repaired by RNase H2-initiated ribonucleotide excision repair (RER). During this process, RNase H2 excises the 5′-side of the incorporated ribonucleotide into DNA [9,10]. Then, Pols δ and ε with PCNA displace the DNA strand with a ribonucleotide, followed by the excision of the flapped DNA by flap endonuclease FEN1 and nick sealing by DNA ligases [11]. RNase H2 enzyme inactivation leads to severe cellular abnormalities, including persistent DNA damage response and p53-mediated apoptosis, which results in embryonic lethality in mice [2,12]. This fatal phenotype can be caused by the alternate false-processing of a ribonucleotide initiated by DNA topoisomerase 1 (Top1). The Top1 cleaves the 3′ of the embedded ribonucleotide and forms a covalent Top1-DNA cleavage complex (Top1cc) at the 3′-terminus [13,14]. The phosphate of Top1cc is susceptible to nucleophilic attack of the 2′-hydroxyl of the ribonucleotide sugar backbone, generating a 2′,3′-cyclic phosphate end and releasing Top1. Then, Top1 cleaves in the 5′-upstream region of the nick containing the 2′,3′-cyclic phosphate, forming a second Top1cc and DNA gap. In yeast, this Top1 processing of a ribonucleotide causes short deletion mutations at tandem repeat sequences [14]. However, such mutations have not been observed in the genomic DNA of RNase H2-deficient mouse-derived cells [7]. The trapped Top1cc also causes DNA replication collapse and DSBs formation [15]. Importantly, it has been reported that Top1-mediated processing of ribonucleotides induces DSBs in yeast and mammalian cells [16,17]. In such cases, the trapped Top1cc with a DSB must be repaired by the downstream repair factors.

In eukaryotes, trapped topoisomerase-DNA complexes are resolved by proteasomal degradation, followed by hydrolysis of the phosphotyrosyl bond between topoisomerases and the DNA backbone by tyrosyl DNA phosphodiesterases (TDPs). TDP1 hydrolyzes the phosphodiester bonds at the 3′-DNA terminus linked to tyrosyl moiety within the trapped Top1cc [18]. In addition, TDP1 has broad substrate specificity for various 3′-blocking lesions resulting from different types of DNA damage and chain-terminating anticancer nucleosides [19–21]. Thus, the biological and clinical importance of the function of TDP1 has been well investigated [22]. TDP2 can resolve 5′-phosphotyrosyl bonds between Top2 and the DNA strand break terminus in Top2cc [23]. In addition to its canonical function, TDP2 also possesses weak cleavage activity for 3′-phosphotyrosyl bonds [24], suggesting the overlapping roles of TDP1 and TDP2 in Top1cc repair [25]. TDP1 and TDP2 are also involved in the repair of topoisomerase-induced DSBs. For example, TDP1 facilitates non-homologous end-joining (NHEJ) pathway via functional interactions with NHEJ factors Ku70/80 and XLF in human cells [26]. In addition, TDP2 is required for efficient NHEJ upon the formation of Top2-mediated DSBs [27].

Based on the biochemical and biological functions of TDP1 and TDP2, these enzymes appear to affect ribonucleotide-induced genomic instability through the processing of Top1-DNA complexes. Based on an *in vitro* study, TDP1 excises the 3′-blocking topoisomerase-derived lesions linked to a single embedded ribonucleotide [28]. However, the disruption of TDP1 in RNase H2-deficient human cells does not exhibit the obvious effect based on growth and survival rates [29]. There is limited evidence supporting the role of TDP1 and TDP2 in mutagenesis of an embedded ribonucleotide in mammalian cells.

We previously reported that a single ribonucleotide induced deletion mutations in the simian virus 40 (SV40)-based shuttle vector that can be propagated in human cells [30]. SV40 DNA replication is carried out by human replication factors including PCNA, Top1, and DNA polymerase δ [31]. Unlike the genome in which DNA replication timing is strictly regulated, the shuttle vector containing SV40 replication origin is rapidly propagated in the presence of SV40 large-T antigen in mammalian cells [32]; the copy number of the vector can reach 100,000 copies per cell within 48 h after transfection. Due to its high replication rate, it is expected that DNA lesions incorporated into the vector could escape from the canonical repair, thereby giving rise to mutation frequencies in the range of 10^−3^ [33,34]. Using the same experimental technique that quantifies the mutagenic properties of an embedded ribonucleotide in cells, we assessed the effect of TDPs deficiency on the mutagenic potential of a single riboguanosine (rG) incorporated into DNA. The disruption of *TDP1* and *TDP2* markedly suppressed the mutagenic potential of an embedded rG. Our results provide *in cellulo* evidence supporting that TDPs mediate mutagenic processing of a ribonucleotide incorporated into DNA rather than protect the DNA against it in mammalian cells, which might contribute to the global genomic instability in RER deficiency. A possible mechanism for the involvement of DNA repair pathways in ribonucleotide-induced mutagenesis is discussed.

## Materials and methods

### Cell culture

The human TSCER2 cell line, derived from human lymphoblastoid TK6 [35], and its derivatives were cultured in RPMI-1640 medium (Nacalai Tesque) supplemented with 10% fetal bovine serum, 200 μg/mL sodium pyruvate, 100 U/mL penicillin, and 100 μg/mL streptomycin at 37°C in an atmosphere of 5% CO_2_ and 100% humidity. *TDP1*^-/-^, *TDP2*^-/-^, and *TDP1*^-/-^/*TDP2*^-/-^ cells were established as previously described [36]. The loss of TDP1 or TDP2 expression in cell lines was confirmed in parallel in a previous study [37].

### Construction of a closed circular double-stranded plasmid DNA site-specifically containing a single ribonucleotide

A plasmid containing a single rG at position 160 of pMY189, the numbering used is that of the *supF* reporter gene in the plasmid with denoting the *Eco*RI site before *supF*, was constructed as previously described [38]. In detail, the 5′-phosphorylated 21-mer primer (5′-CGACTTCGAAGXTTCGAATCC-3′, where X represents dG or rG) was obtained from Tsukuba Oligo Service Co. Ltd. (Ibaraki, Japan). The primer (16 pmol) was annealed with single-stranded DNA (5 μg) of pMY189 that was isolated from JM109 *Escherichia coli* (*E*. *coli*) strain harboring the plasmid using a VCSM13 helper phage. Then, the annealed DNA was incubated with 2 units of T4 DNA polymerase (Sigma) and 250 units of T4 DNA ligase (Takara) at 37°C for 3 h in the reaction mixture in a final volume of 100 μl contained 50 mM Tris-HCl (pH 7.9), 50 mM NaCl, 7 mM MgCl_2_, 1 mM dithiothreitol, 1 mM ATP, 0.5 mM dNTPs, and 12 μg of T4 gene 32 protein (Nippon Gene). The reaction product was treated with 16 units of dam methyltransferase (New England Biolabs) to restore the bacterial methylation pattern. The closed circular double-stranded DNA with dG or rG was purified using ultracentrifugation in a cesium chloride-ethidium bromide gradient. Homogeneity and no degradation of the purified DNA was confirmed by agarose gel electrophoresis before the transfection experiment.

### Forward mutation assay in the *supF* gene

The closed circular double-stranded DNA with or without rG (500 ng) was transfected into TSCER2 cells (2 × 10^6^ cells) using NEPA21 electroporator (Nepa Gene Co. Ltd.) following the manufacturer’s instructions. After incubation for 48 h at 37°C, the replicated plasmid DNA was extracted from cells and treated with *Dpn*I to digest unreplicated plasmids. The resultant DNA was introduced into the KS40/pOF105 indicator *E*. *coli* strain [39]. The transformed cells were plated onto Luria-Bertani (LB) agar selection plates containing nalidixic acid (50 μg/ml), streptomycin (100 μg/ml), ampicillin (150 μg/ml), chloramphenicol (30 μg/ml), 5-bromo-4-chloro-3-indolyl-β-D-galactoside (80 μg/ml), and isopropyl β-D-thiogalactopyranoside (23.8 μg/ml) to select the *E*. *coli* cells harboring a mutated *supF* gene. Then, the cells were plated onto LB titer plates containing ampicillin (150 μg/ml) and chloramphenicol (30 μg/ml) to determine the total number of transformants. The mutant frequency of *supF* was determined based on the numbers of white and faint blue colonies on selection plates and the number of colonies on titer plates. For sequencing analysis to determine the mutation spectrum, the *supF* gene was amplified from mutant colonies by PCR with primers 5′-AGTGCCACCTGACATCTA-3′ and 5′-CAGCAGATTACGCGCAGA-3′. The resulting fragment was treated with ExoSapIT (Thermo Fisher Scientific) to degrade excess primers and deoxynucleoside triphosphates and sequenced using BigDye terminator v3.1 (Thermo Fisher Scientific) on an ABI 3130 genetic analyzer (Thermo Fisher Scientific).

### Determining the recovery ratio of propagated plasmids from human cells

An internal control vector pST189K was constructed by replacing the ampicillin resistance gene and M13 intergenic sequence of pZ189-StuI with the kanamycin resistance gene [40]. The synthetic closed circular DNA containing dG or rG (500 ng) and an equal amount of pST189K (500 ng) were co-transfected into TSCER2 cells (2 × 10^6^ cells) by using NEPA21 electroporator (Nepa Gene Co. Ltd.). After 48 h of incubation, the replicated plasmids were extracted from cells, digested with *Dpn*I, and then introduced into the KS40/pOF105 strain as described above. The transformed cells were plated onto LB plates containing ampicillin (150 μg/ml) or kanamycin (50 μg/ml) in the presence of chloramphenicol (30 μg/ml). The recovery ratio of replicated plasmids was indicated by the number of ampicillin-resistant colonies relative to the number of kanamycin-resistant colonies.

### Statistical analysis

The statistical significance of the *supF* mutant frequency was evaluated using Dunnett’s multiple comparison test. The mutation spectra between the wild-type, *TDP1*^-/-^, *TDP2*^-/-^, and *TDP1*^-/-^/*TDP2*^-/-^ cells were statistically analyzed using Fisher’s exact test with multiple testing corrections using Holm’s method. Statistical significance was indicated by *P-*values of <0.05.

## Results

### Mutagenic events induced by a ribonucleotide embedded into a plasmid in human cells

**Fig 1:**
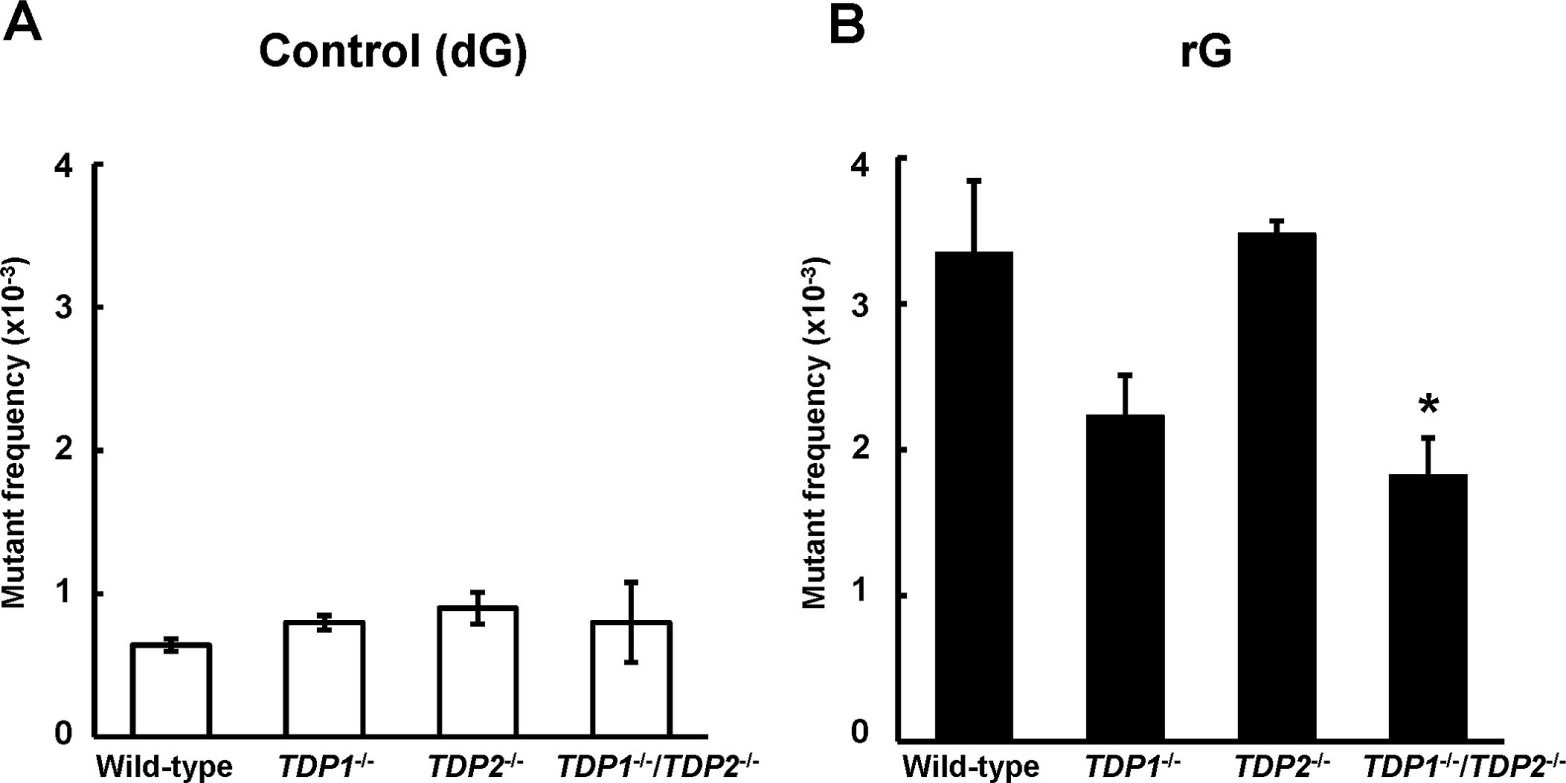
Frequency of *supF* mutants in a shuttle vector containing (A) dG or (B) rG replicated in wild-type (WT), *TDP1*^-/-^, *TDP2*^-/-^, and *TDP1*^-/-^/*TDP2*^-/-^ cells. Mutant frequencies were calculated from the data of S1 Table. Data for control dG are expressed as the mean ± standard error (SE) of at least three independent experiments from two independent clones. Data for rG are expressed as the mean ± SE of three to six independent experiments from two independent clones. *Significant difference between the assessed and WT cells; P < 0.05.

To investigate the mutation induced by a ribonucleotide embedded into DNA, we constructed the shuttle vector site-specifically containing rG and introduced into human TSCER2 cells. The propagated plasmids were extracted from cells, and further introduced into *E*. *coli* KS40/pOF105 indicator strain to determine the *supF* mutant frequency and spectrum. Mutant frequencies were determined as the number of mutant colonies divided by the total number of colonies recovered (S1 Table). In the wild-type cells, the mutant frequency of the plasmid containing rG ((3.4 ± 0.49) × 10^−3^) was significantly higher than that of the control plasmid ((0.64 ± 0.044) × 10^−3^) (P < 0.01) (Fig 1A and 1B). Based on detailed DNA sequencing analysis in the *supF* gene, the most predominant mutations induced by rG were large deletion mutations ranging from 9 to 304 bp in length in the wild-type cells (Table 1 and S2 Table), which was consistent with previous observations [30]. The majority of large deletions were extended to the 5′-upstream region from the position of rG. The second most common mutations were “untargeted” mutations, i.e., base substitution mutations outside the site of rG, which was preferentially observed at guanine bases (Table 1 and S3 Table). In contrast, large deletion mutations were infrequently observed in the control plasmid.

**Table 1:** Mutation spectrum in *supF* induced by a single ribonucleotide in wild-type, *TDP1*^-/-^,*TDP2*^-/-^, and *TDP1*^-/-^/*TDP2*^-/-^ cells.

^0^G^*a*^	Mutation		Wild-type	*TDP1*^-/-^	*TDP2*^-/-^	*TDP1*^-/-^/*TDP2*^-/-^
dG	Mutations at site of ^0^G					
		G:C → T:A	6 (15)	8 (26)	4 (10)	4 (12)
		G:C → A:T	0 (0)	1 (3.0)	1 (3.0)	2 (6.0)
	Large deletion		3 (7.5)	3 (10)	5 (13)	1 (3.0)
	Base substitution, 1–2 bp deletion or insertion outside ^0^G		31 (78)	19 (61)	28 (74)	27 (79)
	Total		40 (100)	31 (100)	38 (100)	34 (100)
rG	Mutations at site of ^0^G					
		G:C → T:A	1 (1.7)	0 (0)	2 (3.0)	3 (5.0)
		G:C → C:G	1 (1.7)	2 (3.0)	3 (4.5)	0 (0)
		G:C → A:T	1 (1.7)	0 (0)	1 (1.5)	0 (0)
	Large deletion		33 (55)	21 (35)	27 (41)	15 (24)**
	Base substitution, 1–2 bp deletion or insertion outside ^0^G		23 (38)	37 (62)	29 (45)	44 (71)
	Other		1 (2.0)	0 (0)	3 (5.0)	0 (0)
	Total		60 (100)	60 (100)	65 (100)	62 (100)

^*a* 0^G indicates the 160th base position in pMY189 where dG or rG was introduced.

^*b*^ Numbers in parentheses represent the percentage of total number of mutants.

** Significant difference between the assessed cells and WT cells; P < 0.01.

### Effect of the disruption of TDP1 and TDP2 on the mutagenic potential of an embedded ribonucleotide

We examined the impact of *TDP1* and *TDP2* deficiency on the mutation induced by a ribonucleotide incorporated into DNA. To compare the *supF* mutant frequency between cell lines, it should be noted that the population doubling times of *TDP1*^-/-^, *TDP2*^-/-^, and *TDP1*^-/-^/*TDP2*^-/-^ cells (14 ± 1.6, 13 ± 0.10, and 14 ± 1.2 h, respectively) were similar to that of the wild-type cells (14 ± 0.66 h). Although the mutant frequency induced by rG was slightly decreased in the absence of *TDP1* ((2.2 ± 0.28) × 10^−3^) compared with the wild-type cells ((3.4 ± 0.49) × 10^−3^), this difference was not statistically significant (Fig 1B). The mutant frequencies in *supF* gene in the control ((0.90 ± 0.11) × 10^−3^) and rG-containing plasmid ((3.5 ± 0.089) × 10^−3^) in *TDP2*^-/-^ cells were comparable to those in the wild-type cells ((0.64 ± 0.044) × 10^−3^ and (3.4 ± 0.49) × 10^−3^, respectively) (Fig 1A and 1B). TDP2 possesses a weak 3′-tyrosyl DNA phosphodiesterase activity, which contributes to resolving Top1-mediated DNA lesions in the absence of TDP1 [24]. Thus, we suspected that the disruption of both TDPs leads to the marked modulation of the mutagenic potential of rG embedded into DNA. For results, the frequency of mutants induced by rG was significantly lower in *TDP1*^-/-^/*TDP2*^-/-^ cells ((1.8 ± 0.26) × 10^−3^) than in the wild-type cells ((3.4 ± 0.49) × 10^−3^) (Fig 1B).

### Deletion mutations caused by an embedded ribonucleotide are suppressed in the absence of TDP1 and TDP2

**Fig 2:**
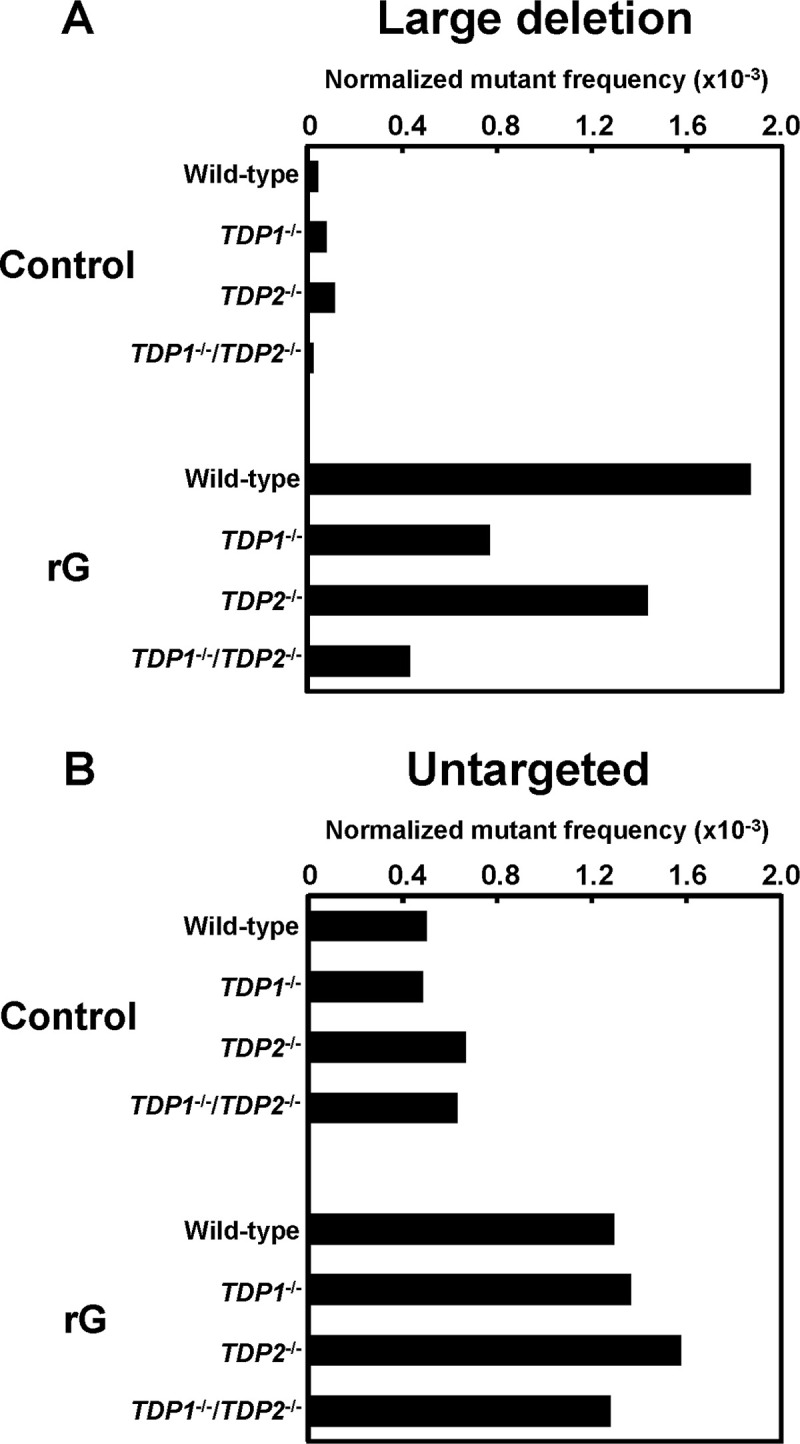
The normalized mutant frequencies of (A) large deletion and (B) untargeted mutations detected in the *supF* gene in the plasmids replicated in the wild-type, *TDP1*^-/-^, *TDP2*^-/-^, and *TDP1*^-/-^/*TDP2*^-/-^ cells. The frequency values of each mutant were calculated based on the number of mutants represented in Table 1. A significant difference was determined based on the number of mutations shown in Table 1.

We also assessed whether the loss of TDPs modulated the mutation spectrum induced by a ribonucleotide in *supF* gene. Among the mutations observed, we focused on large deletion and untargeted mutations that are predominantly induced by rG. In *TDP1*^-/-^ cells, the most predominant mutations induced by rG were large deletion mutations as observed in the wild-type cells (Table 1): the ratio of large deletion mutations in *TDP1*^-/-^ cells (35%) was lower than that in the wild-type cells (55%), although the difference was not significant. Based on the mutant frequency and mutation spectra given in Fig 1B and Table 1, the normalized mutant frequency derived from rG-induced large deletion mutations was calculated to be 0.77 × 10^−3^ in *TDP1*^-/-^ cells, which was approximately 2.4-fold lower than that of the wild-type cells (1.9 × 10^−3^) (Fig 2A). However, the frequency of untargeted mutations was comparable between the wild-type and *TDP1*^-/-^ cells (1.3 × 10^−3^ and 1.4 × 10^−3^, respectively) (Fig 2B). Regardless of the status of *TDP2*, the mutation spectrum in the *supF* gene was not substantially different (Table 1). The normalized frequency of large deletion mutations was slightly decreased by the disruption of *TDP2* (1.4 × 10^−3^) when compared with the wild-type cells (1.9 × 10^−3^) (Fig 2A).

In *TDP1*^-/-^/*TDP2*^-/-^ cells, the ratio of rG-mediated large deletion mutations (24%) was significantly lower than that in the wild-type cells (55%) (Table 1), according to the decreased mutant frequency. The frequency derived from large deletion mutations in *TDP1*^-/-^/*TDP2*^-/-^ cells (0.43 × 10^−3^) was 4.3-fold lower than that in the wild-type cells (1.9 × 10^−3^) (Fig 2A). On the other hand, the frequency of untargeted mutations was comparable between the wild-type and *TDP1*^-/-^/*TDP2*^-/-^ cells (1.3 × 10^−3^ and 1.3 × 10^−3^, respectively) (Fig 2B), suggesting that such untargeted mutations related to rG were not associated with the function of TDP1 or TDP2. Notably, there was no significant difference between the *TDP1*^-/-^/*TDP2*^-/-^ and wild-type cells in the mutant frequency and mutation spectrum in the *supF* gene of the control plasmid (Fig 1A and Table 1).

### Tyrosyl-DNA phosphodiesterases deficiency alters the length of large deletion mutations

**Fig 3:**
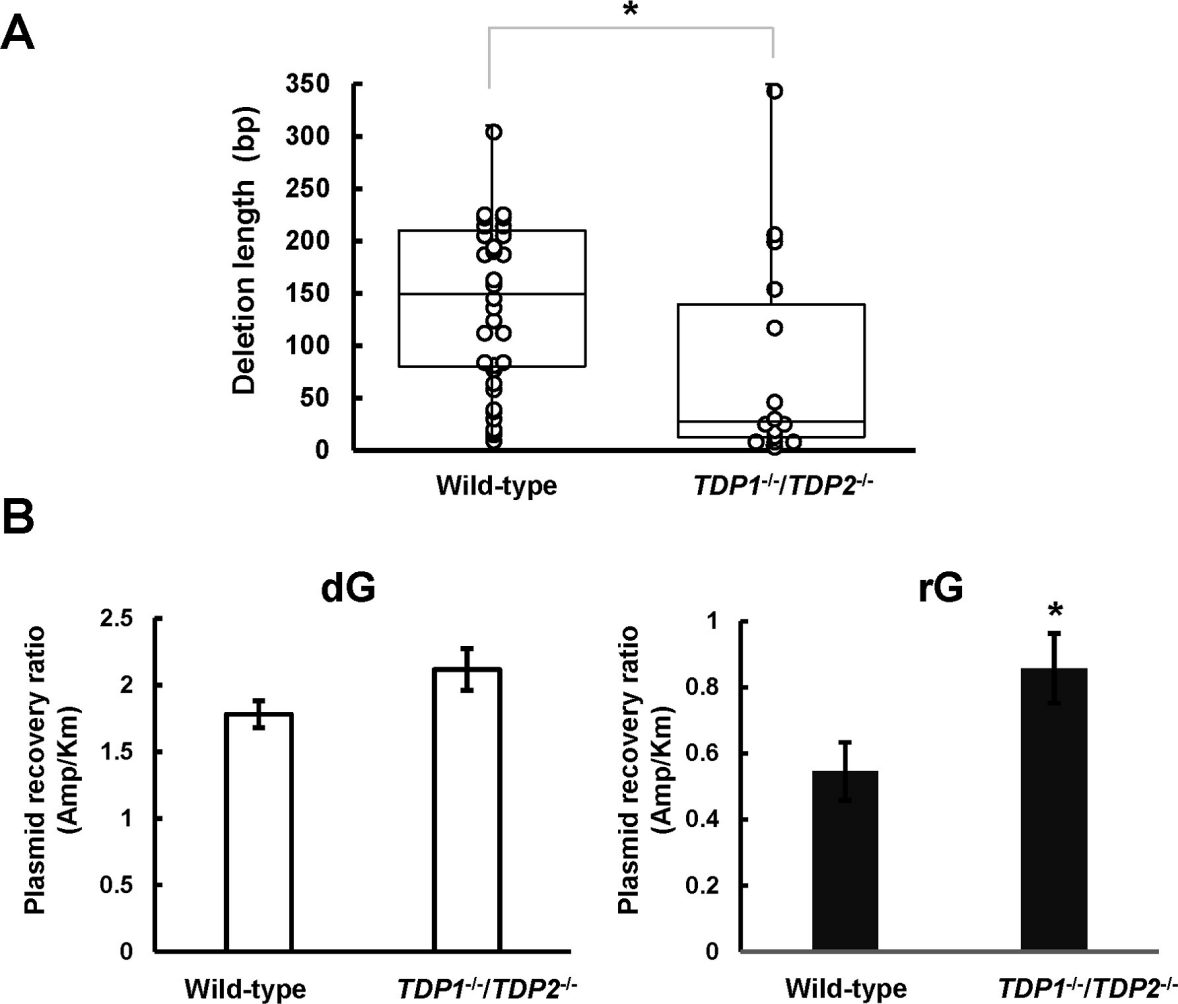
Effect of tyrosyl-DNA phosphodiesterase deficiency on the length of large deletion mutations and the recovery ratio of propagated plasmids. (A) Box plot representing the length of large deletion mutations observed in the indicated genotypes. A significant difference is indicated by an asterisk (*P <0.05). (B) The synthetic closed-circular DNA with or without rG was co-transfected with pST189K into cells. After 48 h of incubation, the replicated plasmids were recovered and introduced into the KS40/pOF105 strain. The transformed *E*. *coli* cells were plated onto LB plates with ampicillin (150 μg/mL) or kanamycin (50 μg/mL) in the presence of chloramphenicol (30 μg/mL). The numbers of ampicillin-resistant colonies relative to those of kanamycin-resistant colonies are presented. Values presented are the mean ± SE from six independent cultures derived from two transfections. A significant difference is indicated by an asterisk (*P <0.05).

To examine whether the loss of both *TDP1* and *TDP2* affects the magnitude of deletions induced by a ribonucleotide, we compared the length of rG-induced large deletion mutations between cells. The median length of large deletion mutations was determined to be 25 bp in *TDP1*^-/-^/*TDP2*^-/-^ cells, which was remarkably shorter than that observed in the wild-type cells (145 bp; Fig 3A). These results raise a question regarding the loss of TDPs affecting the recovery of replicated plasmids from cells resulting from the reduced frequency and length of large deletion mutations. Thus, we compared the relative recovery ratio of propagated plasmids between wild-type and *TDP1*^-/-^/*TDP2*^-/-^ cells. To determine the recovery ratio, the synthetic vector containing dG or rG was co-transfected with an equal amount of the internal control vector pST189K harboring kanamycin-resistant gene. After 48 h incubation, the replicated plasmids were extracted and introduced into the indicator *E*. *coli* strain, which was then plated onto LB plates. The relative recovery ratio was calculated from the numbers of ampicillin-resistant colonies relative to kanamycin-resistant colonies. The recovery ratio of replicated plasmids after introducing the shuttle vector containing rG (0.55 ± 0.087) was lower than that related to dG (1.8 ± 0.10) in the wild-type cells. Interestingly, we found that the recovery ratio after the transfection of the shuttle vector with rG increased with disruption of TDPs (0.86 ± 0.11) (Fig 3B), suggesting that TDPs-dependent processing of an embedded ribonucleotide is mutagenic and compromises the plasmid propagation.

## Discussion

The impact of ribonucleotide accumulation into the genome has been investigated *in vitro* and *in vivo* [1,41,42], and the false processing of incorporated ribonucleotides has been found to lead to genomic instability [14,16,17]. Despite extensive studies on the role of TDPs in genome stability, little is known about the involvement of TDPs in the processing of the ribonucleotide incorporated into DNA in mammalian cells. In principle, disruption of the canonical RER is considered to be required to assess the mutagenic processing initiated by Top1. RER-deficient mammalian cells, however, are viable only in the absence of the p53 tumor suppressor gene. The loss of p53 causes another problem whereby the background mutation rate is dramatically increased because of the roles of p53 in genome integrity [43], which could complicate the interpretation of results [44]. To specifically visualize the mutagenic events at an embedded ribonucleotide that is readily removed by RER, we employed the shuttle vector system that is rapidly propagated in cells, in which some extent of an embedded ribonucleotide might escape the canonical repair in RER-proficient TK6 cells. Based on our results, a single embedded rG induced predominantly large deletion mutations in which the deleted regions were extended to the 5′-direction from the site of rG in the *supF* gene (S2 Table). In cells, the mutagenic processing of an embedded ribonucleotide is initiated by the cleavage at the 3′-side of a ribonucleotide, generating a 3′-dirty DNA end containing 2′,3′-cyclic phosphate through the formation of Top1cc. Then, the second Top1cc is formed at 5′-upstream of the nick, generating the gap [45]. Because Top1cc is also associated with the formation of DSBs upon DNA replication in cells [16,17], the deletion mutations observed in this study can be the result of these Top1-mediated fault processing events.

TDP1 has an important role of tolerating DNA damage resulting from the formation of Top1cc [28]. When investigating the frequency of each type of mutations, the *TDP1* deficiency caused a 2.4-fold decrease in the frequency of large deletion mutations (Fig 2A). This moderate effect observed in *TDP1*^-/-^ cells is possibly explained by the contribution of TDP2 activity to resolve Top1-induced DNA damage in the absence of TDP1 [24]. In addition, the disruption of both *TDP1* and *TDP2* resulted in a significant reduction of the rG-induced large deletion mutations (Fig 1B and Table 1). Thus, our results suggest that TDPs mediate the mutagenic processing of a ribonucleotide incorporated into DNA.

Importantly, *TDP1* and *TDP2* deficiencies decreased the length of rG-mediated deletion mutations (Fig 3A). In detail, most rG-induced large deletion mutations (27/33) were longer than 50 bp, and the remaining (6/33) were smaller deletions (<50 bp) in the wild-type cells (S2 Table). A fraction of >50 bp deletion mutations (5/15) decreased in *TDP1*^-/-^/*TDP2*^-/-^ cells, whereas smaller deletions ranging from 3 to 50 bp were still evident (10/15) (S2 Table). Consistent with the reduced length of the deletion mutations, the recovery ratio of propagated plasmids related to rG was enhanced in *TDP1*^-/-^/*TDP2*^-/-^ cells (Fig 3B). These results imply that the distinct repair pathway can play a complementary role in the TDPs-dependent mutagenic pathway in cells. Recently, it was reported that human AP endonuclease 2 (APE2) catalyzes 3′-5′ nucleolytic resection at the Top1cc terminus and 2′,3′-cyclic phosphate-DNA end [29]. The exonuclease activity of APE2 is essential for the activation of ATR-Chk1 DNA damage-response [46]. The ATR-Chk1 pathway regulates DNA replication fork speed and prevents further DNA damage [47,48]. Therefore, the APE2-mediated processing cascade can be crucial for suppression of the ribonucleotide-induced mutagenesis. Obviously, the loss of APE2 or ATR results in a synthetic lethal phenotype in RNase H2 deficiency, but cells lacking both TDP1 and RNase H2 are viable [29,49], suggesting that APE2 plays a pivotal role in protecting the genome in the absence of the canonical RER. Consistent with these studies, the yeast homologue of APE2 suppresses Top1cc-mediated mutations in RER-deficient cells [50]. It is noted that a substantial number of rG-induced smaller deletions has been observed in the absence of TDPs. These mutations might be mediated by a distinct machinery such as XPF-ERCC1 that is involved in the repair of Top1-induced DNA damage via formation of DSB [51].

In contrast, it has been proposed that TDPs are involved in repairing stalled topoisomerase-DNA complexes in conjunction with the NHEJ pathway in mammalian cells [26,27]. Upon the formation of Top1-induced DSBs, TDP1 is phosphorylated by ATM and DNA-dependent protein kinase (DNA-PK) [52]. TDP1 also promotes DNA binding by the initial NHEJ components Ku70/80 and XLF and stimulates the activity of DNA-PK, facilitating the efficient NHEJ-mediated DSB repair [26]. Consistently, the depletion of TDP1 reduces NHEJ efficiency in cells [53]. An earlier study reported that the lack of the NHEJ pathway confers resistance to camptothecin, a selective Top1 inhibitor that stabilizes Top1cc, in chicken DT40 cells [15]. Thus, in a similar manner, the disruption of the TDPs-dependent NHEJ pathway might alleviate the mutagenic consequences of ribonucleotide processing through an alternate repair such as the APE2-dependent pathway. Notably, a question arises about how large deletion mutations occur as the consequence of the TDP-dependent pathway. Although its detail mechanism remains unclear, a recent study reported that similar large deletion mutations were induced by the trapped Top1cc in yeast [54]. Based on our results, most deletion mutations were extended to the 5′-upstream region from the site of an embedded ribonucleotide (S2 Table). Because the frequency of these mutations was remarkably decreased by the depletion of TDPs (Fig 2A), such mutations might have been induced via the formation of Top1cc 5′-upstream of the ribonucleotide. Based on *in vitro* studies, however, the second Top1cc is formed only a few bases upstream from the nick [14,28,45]. The second Top1-cleavage could possibly occur over a range of longer nucleotides in cellular circumstances, resulting in large deletion mutations [54]. Alternatively, the cleavage of 3′-blocking lesions by TDPs may be followed by further resection reaction in certain situations.

**Fig 4:**
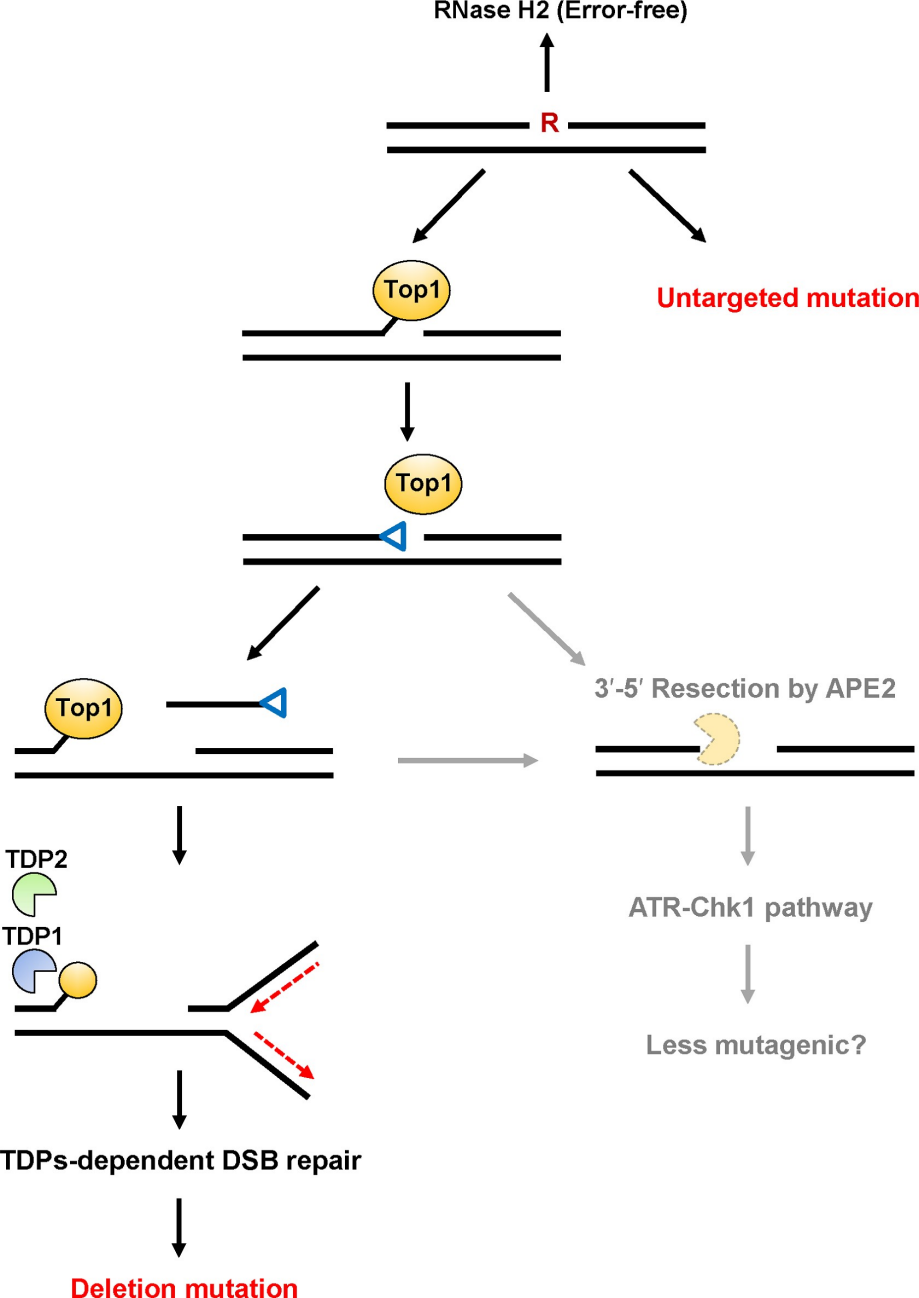
Predicted model of mutagenic events induced by a ribonucleotide incorporated into DNA. The incorporated ribonucleotide is primarily repaired by RNase H2-initiated RER in an error-free manner. Alternate processing of the embedded ribonucleotide is initiated by Topoisomerase 1 (Top1)-mediated cleavage at the 3′-side of the ribonucleotide to generate a transient Top1-DNA cleavage complex (Top1cc). Then, the nucleophilic attack of the 2′-hydroxyl group to the phosphotyrosyl bond can release Top1 with the formation of 2′,3′-cyclic phosphate (depicted as a blue triangle). This 3′-blocking lesion can be processed by possible pathways as described below. A second Top1cc formation leads to the release of a DNA fragment containing 2′,3′-cyclic phosphate and the formation of a DNA gap. During the excision of a second Top1cc by TDP1 and TDP2, DNA replication fork stalling is induced by the Top1cc adduct, resulting in the formation of DSBs. The following NHEJ repair pathway might result in the formation of deletion mutations. Alternatively, the DNA terminus containing 2′,3′-cyclic phosphate and a second Top1cc adduct is subjected to resection catalyzed by the exonuclease, AP endonuclease 2 [29], which might be performed in an more error-free manner via the activation of ATR-Chk1 pathway. Untargeted base substitution mutations are induced by a ribonucleotide embedded into DNA through the TDP-independent mechanism.

By taking together our data and those of other studies, we proposed a model of ribonucleotide-induced mutagenesis in mammalian cells (Fig 4). The formation of 2′,3′-cyclic phosphate by Top1-mediated cleavage of a ribonucleotide is followed by a second Top1cc formation, leaving a DNA gap between the 3′-Top1cc and 5′-hydroxyl termini. The Top1cc-induced DSB via replication fork collapse can be repaired by the TDPs-dependent NHEJ pathway. Then, deletion mutations might be formed as a result of the unfavorable DSB repair. Alternatively, the other distinct factor, APE2, is involved in the resection of a trapped Top1cc-derived adduct and the 2′,3′-cyclic phosphate-DNA end, which may suppress the mutagenic consequences of ribonucleotide processing via the activation of ATR-Chk1 pathway.

Interestingly, a substantial amount of untargeted mutations were detected in the *supF* gene derived from plasmids containing rG in all cell lines (Fig 2B and Table 1). It was unexpected to observe that such mutations were preferentially induced at guanine sequences (S3 Table). Although the mechanistic insight of these mutations is unclear, an unrepaired ribonucleotide in the template DNA might influence the fidelity of local DNA replication through unknown mechanism (Fig 4).

Overall, we provided evidence that mutagenesis induced by an embedded ribonucleotide is mediated by TDP1 and TDP2 in human cells. In mammals, loss of the canonical RER is associated with the risk of prostate adenocarcinoma, glioma, and colorectal tumor due to the large scale genomic instability [7,49,55]. The TDPs-dependent repair cascade may therefore contribute to the tumorigenesis in RER-deficient cells. If so, such alternate repair pathway can be a candidate target for cancer therapy. Further investigations are needed to thoroughly understand the impact of the processing pathway for the accumulated ribonucleotides in the mammalian genomic DNA.

## Supporting information

S1 Table Mutant frequencies of the supF gene calculated from the number of bacterial colonies. (XLSX)

S2 Table Sequences of large deletion mutations induced by a single ribonucleotide in supF. (XLSX)

S3 Table Untargeted mutations detected in supF. (XLSX)
